# Effects of a Formula Containing Two Types of Prebiotics, Bifidogenic Growth Stimulator and Galacto-oligosaccharide, and Fermented Milk Products on Intestinal Microbiota and Antibody Response to Influenza Vaccine in Elderly Patients: A Randomized Controlled Trial

**DOI:** 10.3390/ph8020351

**Published:** 2015-06-18

**Authors:** Shinya Nagafuchi, Taketo Yamaji, Akihiro Kawashima, Yukiko Saito, Takeshi Takahashi, Takayuki Yamamoto, Mitsuo Maruyama, Hiroyasu Akatsu

**Affiliations:** 1 Food Science Research Labs., R&D Division, Meiji Co., Ltd., Odawara, Kanagawa 250-0862, Japan; E-Mails: shinya.nagafuchi@meiji.com (S.N.); taketo.yamaji@meiji.com (T.Y.); akihiro.kawashima@meiji.com (A.K.); takeshi.takahashi@meiji.com (T.T.); 2 Choju Medical Institute, Fukushimura Hospital, Toyohashi, Aichi 441-8124, Japan; E-Mails: ytoyama@chojuken.net (Y.S.); ta_yamamoto@fukushimura.net (T.Y.); 3 Department of Mechanism of Aging, Research Institute, National Center for Geriatrics and Gerontology, Obu, Aichi 474-8522, Japan; E-Mail: michan@ncgg.go.jp; 4 Department of Medicine for Aging in Place and Community-Based Medical Education, Nagoya City University Graduate School of Medical Sciences, Nagoya, Aichi 467-8601, Japan

**Keywords:** enterally fed patients, *Bifidobacterium*, hemagglutination inhibition test, seroprotective rate

## Abstract

We investigated the effect of a formula containing two different prebiotics (bifidogenic growth stimulator and galacto-oligosaccharide) and fermented milk products on intestinal microbiota and antibody responses to an influenza vaccine in enterally fed elderly in-patients. Patients were administered either formula containing prebiotics and fermented milk products (group F: *n* = 12, 79.9 ± 9.5 years old) or standard formula (group C: *n* = 12, 80.7 ± 10.1 years old) via percutaneous endoscopic gastrostomy during a 14-week intervention period. Subjects were immunized with an influenza vaccine (A/H1N1, A/H3N2, and B) at week 4 of the intervention. Blood biochemical indices, intestinal bacteria populations and antibody titers were analyzed. *Bifidobacterium* counts increased significantly in group F compared with group C. The enhanced antibody titers against A/H1N1 were maintained in group F for a longer period compared with group C. The titers against A/H3N2 were unchanged between both groups, and those against B were significantly lower in group F than in group C, although few subjects had seroprotective titers against A/H3N2 and B. These results suggest that administration of the formula containing prebiotics and fermented milk products may maintain antibody titers for longer periods through the improvement of intestinal microbiota.

## 1. Introduction

The elderly are generally known to be at greater risk of infectious diseases, as their immune responses are less vigorous than in younger adults. Influenza causes morbidity and mortality, particularly among older adults [1]. Influenza vaccination protects against influenza virus infection, reducing the risks of death and hospitalization associated with serious complications, such as pneumonia [2,3], however, the age-related decline of immune functions also reduces the responses of elderly patients to the influenza vaccine [4]. Taking these studies into consideration, it is very important to enhance the vaccine responses in the elderly.

Prebiotics and probiotics have been investigated for their usefulness in a range of clinical areas [5,6], including prevention of influenza virus infection [7]. For example, Vos *et al.* have suggested that consumption of dietary oligosaccharides, such as galacto-oligosaccharide (GOS), may augment systemic Th1-dependent immune responses in a murine vaccination model through the enhancement of a proportion of fecal bifidobacteria and lactobacilli [8]. Influenza-vaccinated healthy elderly subjects who consumed milk fermented with *Lactobacillus* had enhanced antibody titers against the virus [9].

On the other hand, a new type of prebiotic, known as bifidogenic growth stimulator (BGS), is a product of milk whey protein fermented by *Propionibacterium freudenreichii* ET-3 isolated from Swiss cheese. BGS was shown to selectively stimulate the growth of a *Bifidobacterium* spp. without a growth stimulating effect on other intestinal bacteria [10,11].

In our previous intervention study, we examined the effects of simultaneous administration of BGS, GOS, and an enteral formula containing fermented milk products on the intestinal microflora and acquired immunity after influenza vaccination in enterally fed elderly patients [12]. These results suggested that simultaneous administration of these three nutrients might improve the intestinal microflora, contributing to longer-term maintenance of enhanced antibody titers against vaccines. However, in our previous study, serum nutritional indices such as total protein (TP) and albumin (Alb) were significantly higher in the intervention group than the control group during study period [12].

Antibody responses to vaccine antigens are reduced in the undernourished elderly population. The antibody responses to influenza vaccination in hospitalized elderly patients were related to nutritional indices such as serum albumin levels [13]. Therefore, in this randomized controlled trial, we examined the effects of an enteral formula containing two different types of prebiotics, BGS and GOS, and fermented milk products on the intestinal microflora and antibody titers after influenza vaccination in enterally fed elderly patients with no differences in baseline blood nutritional indices.

## 2. Experimental Section

### 2.1. Enteral Formula and Prebiotics

Two types of enteral formula, a standard enteral formula (Meibalance^®^; Meiji Co., Ltd., Tokyo, Japan) and a study formula containing prebiotics and fermented milk products (YH-Flore; Meiji Co., Ltd.) were used. Both types of formulae were cow-milk based. The standard formula contained casein and whey proteins. The study formula contained two different types of prebiotics, BGS (1.65 μg/100 kcal as 1,4-dihydroxy-2-naphthoic acid (DHNA), the active substance of BGS) and GOS (0.4 g/100 kcal), and pasteurized milk products fermented by *Lactobacillus delbrueckii* subsp. *bulgaricus* and *Streptococcus thermophilus*. The study formula contained essentially the same nutrients as the standard formula, with the exceptions of selenium, manganese, vitamin A, and vitamin C (Table 1).

**Table 1:** Nutrients in the standard and study enteral formulae.

Nutrients	Ingredients	Units	Amount per 100 kcal	
			Standard Formula	Study Formula
Prebiotics	BGS (DHNA) *	μg	-	1.65
	Galacto-oligosaccharide	g	-	0.4
Macronutrients	Protein	g	4.0	4.0
	Fat	g	2.8	2.8
	Carbohydrate	g	14.5	14.4
	Fiber	g	1.0	1.5
Vitamins	A	μgRE **	60	114
	D	μg	0.5	0.5
	E	mg	3.0	3.0
	K	μg	5.0	1.8
	B_1_	mg	0.15	0.15
	B_2_	mg	0.2	0.2
	Niacin	mg	1.6	1.6
	B_6_	mg	0.3	0.3
	B_12_	μg	0.6	0.6
	Folic acid	μg	50	50
	Biotin	μg	15	7.5
	Pantothenic acid	mg	0.6	0.6
	C	mg	16	50
Electrolytes	Na	mg	110	100
	K	mg	100	100
	Ca	mg	60	80
	Mg	mg	20	20
	P	mg	60	85
	Cl	mg	140	110
Minerals	Fe	mg	1.0	1.0
	Zn	mg	0.80	1.0
	Cu	mg	0.08	0.05
	Mn	mg	0.23	0.01
	Cr	μg	3.0	2.4
	Mo	μg	2.5	2.9
	Se	μg	3.5	6.0
	I	μg	15	12.7

* Study formula contains BGS (1.65 μg/100 kcal as 1,4-dihydroxy-2-naphthoic acid (DHNA), the active substance of BGS). ** RE, retinol equivalents.

### 2.2. Study Design and Participants

This study was an open-label, randomized, controlled trial that was carried out according to the Helsinki Declaration. This study was registered under the UMIN identifier NO. UMIN000013593. The study protocol was approved by the ethical committees of the Fukushimura Hospital and Meiji Co., Ltd. In this study, written informed consent was obtained from all subjects or their relatives. The patients’ anonymity has been preserved. Elderly patients (> 60 years old) on enteral nutrition hospitalized at Fukushimura Hospital were enrolled as subjects. The exclusion criteria were patients with type 1 diabetes, type 2 diabetes, chronic kidney disease, severe infection, autoimmune disease, immunodeficiency, hepatic failure, cow's milk allergy, soybean allergy, or gastrointestinal disorders such as diarrhea. Thirty-two elderly subjects or their relatives consented.

At the start of this study (week 0), subjects were randomly assigned to a control group (group C: 16 subjects) or an intervention group (group F: 16 subjects). The standard enteral formula and the study enteral formula were used for the control and intervention groups, respectively. These formulae were administered to elderly patients by percutaneous endoscopic gastrostomy (PEG) during the 14-week intervention period from November 2010 to February 2011. Following the intervention period, standard formula was enterally fed in both groups for a 4-week follow-up period from February 2011 to March 2011. During the 14-week intervention period, four subjects in group C and four subjects in group F dropped out of because of transition from enteral nutrition to parenteral nutrition. Overall, 12 subjects in each group completed the study. Two subjects (one subject in each group) were excluded from the analysis of antibody titers because they were vaccinated on a different schedule from the study design. The influenza vaccine was administered subjects in each group at week 4 of this study. Stool samples were collected at weeks 0, 4, 8, 12, and 18. Blood samples were collected at weeks 0, 4, 6, 8, 12, and 18.

### 2.3. Antibody Titers against Influenza Vaccine Antigens

The influenza vaccine was administered subcutaneously in the upper arm of subjects at week 4. Viral strains of the vaccine were A/California/7/2009 (A/H1N1), A/Victoria/210/2009 (A/H3N2), and B/Brisbane/60/2008 (B). The hemagglutinin content of each strain was ≥ 30 μg/strain. Biochemical markers were measured. Plasma antibody titers against A/H1N1, A/H3N2, and B were measured by the hemagglutination inhibition test (HI test). An HI antibody titer ≥ 40 was regarded as seroprotective [14,15]. Subjects who were seroprotected before vaccination (at week 4) were excluded from the analysis of the seroprotective rate (%) and HI antibody titers after vaccination. Analysis of the seroprotective rate and HI antibody titers were performed in the subjects who were seronegative (titer < 40) before vaccination (at week 4). The seroprotective rate (%) was calculated as follows:

Seroprotective rate (%) = 100 × (Number of seroprotected subjects)/(Number of seronegative subjects before vaccination)
(1)

### 2.4. Biochemical Indices

Serum nutrition indices, such as total protein (TP) and albumin (Alb), were assayed by the biuret and bromocresol green (BCG) methods, respectively. Prealbumin (p-Alb) and transferrin (Tf) levels were measured by the N-assay TIA prealbumin NITTOBO (Nittobo Medical, Tokyo, Japan) and N-Assay TIA Tf-H NITTOBO (Nittobo Medical), respectively. Total cholesterol and LDL-cholesterol levels were measured by N-Assay L T-CHO-H NITTOBO (Nittobo Medical) and Cholestest LDL (Sekisui Medical, Tokyo, Japan), respectively. All measurements were performed by the Tousan Labo Center (Toyohashi, Aichi, Japan).

### 2.5. Microbiota

We have measured seven major kinds of bacteria and total bacteria in feces. Bacterial counts were determined by real-time PCR (described below). The collected stool samples were quickly stored at −20 °C. Fecal bacteria gene analysis was performed as described below; fecal microbial DNA was extracted from stool samples using the QIAamp DNA stool mini kit (Qiagen, Tokyo, Japan) and multi-beads shocker (Yasui Kikai Inc., Osaka, Japan). In addition, counts were determined for each bacterium, *Bifidobacterium*, *Bacteroides*, *Clostridium coccoides*, *Clostridium leptum* [16], *Lactobacillus* [17], *Enterobacteriaceae* [18], *Enterococcus* and total bacteria [19] with the ABI 7300 real-time PCR system (Applied Biosystems, Tokyo, Japan) using QuantiTect SYBR Green PCR kit (Qiagen) and published primer base sequences and reaction conditions.

The primers used to enumerate the target bacterial groups in the fecal samples are shown in Table 2. The amplification program consisted of one cycle of reverse transcriptase denaturing at 95 °C for 15 min, followed by 40–55 cycles of denaturation at 94 °C for 15 s, annealing at each temperature (Table 2) for 30 s, and extension at 72 °C for 30 s. The fluorescent products were detected at the end of the extension step of each cycle. The measured count of each bacteria per 1 g feces was log-transformed [log_10_ (count/g of feces)].

**Table 2:** Primer sets used in this study.

Target Organism		Sequence (5' to 3')	Annealing
			Temperature (°C)
*Bifidobacterium*	Forward	CTCCTGGAAACGGGTGG	60
	Reverse	GGTGTTCTTCCCGATATCTACA	
*Lactobacillus*	Forward	CTTGTACACACCGCCCGTCA	57
	Reverse	CTCAAAACTAAACAAAGTTTC	
*Bacteroides*	Forward	ATAGCCTTTCGAAAGRAAGAT	50
	Reverse	CCAGTATCAACTGCAATTTTA	
*C. coccoides*	Forward	AAATGACGGTACCTGACTAA	50
	Reverse	CTTTGAGTTTCATTCTTGCGAA	
*C. leptum*	Forward	GCACAAGCAGTGGAGT	50
	Reverse	CTTCCTCCGTTTTGTCAA	
*Enterobacteriaceae*	Forward	CATTGACGTTACCCGCAGAAGAAGC	58
	Reverse	CTCTACGAGACTCAAGCTTGC	
*Enterococcus*	Forward	CCCTTATTGTTAGTTGCCATCATT	56
	Reverse	ACTCGTTGTACTTCCCATTGT	
Total bacteria	Forward	ACTCCTACGGGAGGCAGCAGT	60
	Reverse	GTATTACCGCGGCTGCTGGCAC	

### 2.6. Data Analysis

For demographic and anthropometric characteristics, nutrient intakes, biochemical indices, and intestinal microbiota, the Student’s *t*-test of equal variance or the Welch test of unequal variance was applied for comparison between the two groups. Antibody titers were measured by the HI test, and significant differences between the two groups were analyzed by the Mann–Whitney *U* test. Analysis after vaccination was performed using the Friedman test. Statistically significant differences in the seroprotective rates between the two groups were investigated by Fischer’s exact probability test. Differences of *p* < 0.05 were considered to be significant.

## 3. Results

### 3.1. Safety of Formulae

There were no important harmful or unintended effects observed in groups C or F during the study period.

### 3.2. Demographic and Anthropometric Characteristics, Nutrient Intakes and Blood Biochemical Indices of Subjects

The clinical characteristics of the subjects are shown in Table 3. No significant differences in sex, age, weight, or BMI were observed between groups C and F. Energy, protein, fat, and carbohydrate intakes per day were similar between both groups (Table 3).

**Table 3:** Characteristics and nutrient intakes of subjects at the start of the study.

Characteristics	Group C	Group F	*P* value
Number of subjects	12	12	-
Male/Female	6/6	5/7	N.S.
Age (years)	80.7 ± 10.1	79.9 ± 9.5	N.S.
Weight (kg)	43.0 ± 5.7	41.9 ± 8.0	N.S.
BMI	17.8 ± 2.5	17.6 ± 3.3	N.S.
Nutrient intakes			
Energy (kcal/kg/day)	23.5 ± 3.0	24.5 ± 5.6	N.S.
Protein (g/kg/day)	0.9 ± 0.1	1.0 ± 0.2	N.S.
Fat (g/kg/day)	0.7 ± 0.1	0.7 ± 0.2	N.S.
Carbohydrate (g/kg/day)	3.4 ± 0.4	3.5 ± 0.8	N.S.

Values are presented as means ± standard deviation.

Table 4 shows the levels of the blood biochemical markers. There were no significant differences in TP, p-Alb, Tf, total cholesterol (TC), LDL-cholesterol (LDL-c) levels, or immunoglobulin between the two dietary groups during the study period. A significant difference in Alb levels was observed between the two dietary groups at week 12.

**Table 4:** Biochemical indices of subjects (*n* = 12).

Biochemical Indices	Group	Week 0	Week 4	Week 6	Week 8	Week 12
IgG (g/dL)	Group C	1.7 ± 0.6	1.7 ± 0.5	1.8 ± 0.5	1.8 ± 0.5	1.8 ± 0.5
	Group F	1.6 ± 0.5	1.6 ± 0.4	1.6 ± 0.4	1.6 ± 0.5	1.6 ± 0.5
IgA (mg/dL)	Group C	372 ± 139	373 ± 140	390 ± 143	384 ± 146	401 ± 153
	Group F	415 ± 139	400 ± 107	401 ± 110	404 ± 131	415 ± 118
IgM (mg/dL)	Group C	95.5 ± 46.6	96.3 ± 50.6	99.2 ± 51.5	102.0 ± 50.3	103.4 ± 54.8
	Group F	80.4 ± 36.1	80.8 ± 38.8	77.1 ± 36.0	82.3 ± 36.2	85.5 ± 33.0
Albumin (g/dL)	Group C	3.2 ± 0.4	3.2 ± 0.4	3.3 ± 0.3	3.3 ± 0.3	3.1 ± 0.4
	Group F	3.3 ± 0.3	3.5 ± 0.4	3.4 ± 0.3	3.4 ± 0.3	3.4 ± 0.2 *
Total protein (g/dL)	Group C	6.6 ± 0.5	6.7 ± 0.5	6.8 ± 0.7	6.8 ± 0.7	6.9 ± 0.6
	Group F	6.6 ± 0.9	7.0 ± 0.6	6.7 ± 0.5	6.6 ± 0.7	7.2 ± 0.6
Prealbmin (mg/dL)	Group C	18.9 ± 5.0	18.1 ± 6.4	17.9 ± 4.9	19.5 ± 6.2	19.2 ± 5.8
	Group F	18.8 ± 4.9	18.9 ± 7.5	19.1 ± 6.2	20.1 ± 5.1	22.1 ± 5.6
Transferrin (mg/dL)	Group C	213 ± 40	207 ± 45	217 ± 43	223 ± 49	222 ± 41
	Group F	205 ± 35	205 ± 33	208 ± 28	221 ± 22	227 ± 32
Total cholesterol	Group C	188 ± 39	189 ± 45	189 ± 37	190 ± 39	190 ± 37
(mg/dL)	Group F	188 ± 40	211 ± 35	203 ± 40	207 ± 37	219 ± 42
LDL-cholesterol	Group C	115 ± 30	118 ± 34	114 ± 30	118 ± 33	123 ± 31
(mg/dL)	Group F	111 ± 28	129 ± 22	116 ± 29	123 ± 29	134 ± 29

Values are presented as means ± standard deviation. * Significant differences between groups C and F (*p* < 0.05).

Biochemical indices at the follow-up period (week 18) were similar between both groups (data not shown).

### 3.3. Microbiota

Fecal bacterial counts [log_10_ (counts/g of feces)] are shown in Table 5. There were no significant differences between the two groups at week 0 in the counts of seven major kinds of fecal bacteria or total bacteria. *Bifidobacterium* counts in group F increased gradually and were significantly higher than those in group C at weeks 8 and 12. The differences in *Bifidobacterium* counts between two groups remained statistically significant at the 4-week follow-up (week 18).

Although there was little difference in the average *C. coccoides* counts between groups C and F, at week 12 *C. coccoides* counts in group F were significantly lower than in group C. There were no significant differences between the two groups during the study period in any other fecal or total bacterial counts. All other fecal bacterial counts, except for *Bifidobacterium,* were similar between both groups at the 4-week follow-up (week 18).

**Table 5:** Fecal bacterial counts in groups C and F.

Bacteria	Group	Week 0	Week 4	Week 8	Week 12	Week 18
*Bifidobacterium*	Group C	4.4 ± 3.4	2.9 ± 3.7	3.4 ± 3.3	4.4 ± 2.2	4.0 ± 2.1
	Group F	5.6 ± 2.7	6.1 ± 3.9	6.9 ± 3.5 *	8.0 ± 2.6 *	6.6 ± 2.5 *
*Lactobacillus*	Group C	5.9 ± 2.6	5.9 ± 3.1	5.9 ± 3.1	5.7 ± 3.0	4.9 ± 3.3
	Group F	5.9 ± 3.3	6.4 ± 2.8	5.9 ± 2.9	6.6 ± 3.0	7.0 ± 2.0
*Bacteroides*	Group C	10.4 ± 0.5	10.6 ± 0.4	9.9 ± 0.8	10.4 ± 0.5	10.5 ± 0.6
	Group F	10.0 ± 0.7	10.8 ± 0.6	10.3 ± 1.1	10.1 ± 1.0	10.3 ±0.6
*C. coccoides*	Group C	10.8 ± 0.3	10.9 ± 0.3	10.7 ± 0.5	10.9 ± 0.2	10.9 ± 0.3
	Group F	10.8 ± 0.4	10.8 ± 0.3	10.7 ± 0.4	10.6 ± 0.4*	10.8 ± 0.3
*C. leptum*	Group C	10.4 ± 0.2	10.3 ± 0.2	10.3 ± 0.4	10.4 ± 0.2	10.3 ± 0.5
	Group F	10.0 ± 0.9	10.2 ± 0.3	10.1 ± 0.4	10.2 ± 0.4	10.4 ± 0.2
*Enterobacteriaceae*	Group C	8.3 ± 0.9	8.1 ± 1.0	7.5 ± 1.6	7.7 ± 1.4	7.4 ± 1.6
	Group F	8.0 ± 1.3	8.7 ± 1.4	8.5 ± 1.1	8.2 ± 1.3	8.0 ± 1.3
*Enterococcus*	Group C	6.3 ± 1.3	6.0 ± 1.4	6.1 ± 1.3	6.7 ± 1.4	6.4 ± 1.3
	Group F	7.0 ± 0.9	6.7 ± 1.1	6.6 ± 1.1	6.8 ± 1.1	6.8 ± 1.7
Total bacteria	Group C	11.4 ± 0.2	11.4 ± 0.2	11.2 ± 0.4	11.5 ± 0.2	11.4 ± 0.3
	Group F	11.4 ± 0.3	11.6 ± 0.2	11.4 ± 0.3	11.5 ± 0.3	11.5 ± 0.1

Values are presented as means (log_10_ count/g of feces) ± standard deviation. * Significant differences between groups C and F (*p* < 0.05).

### 3.4. Antibody Titers against Influenza Vaccine

The number of patients who achieved seroprotective antibody titers, the seroprotective rate (%) and the HI antibody titers are shown in Table 6. Influenza vaccines were administered at week 4 in each group.

Analysis of the number of seroprotective patients, the seroprotective rate (%) and HI antibody titers were performed in the subjects who were seronegative (titer < 40) before vaccination. Since there were three subjects (group C: two subjects; group F: one subject) with seroprotective antibody titers against the B antigen (titer ≥ 40) before vaccination, these subjects were excluded from analysis of the number of seroprotective patients, the seroprotective rate, and HI antibody titers against the B antigen. No subjects had seroprotective antibody titers against the A/H1N1 or A/H3N2 antigens before vaccination.

**Table 6:** The number (rate (%)) of seroprotective patients (**upper number**) and antibody titers (**lower number**).

Antigen	Group	The Number (Rate (%)) of seroprotective patients (HI ≥ 40) HI antibody titers				
		Week 0	Week 4 vaccination	Week 6	Week 8	Week 12
A/H1N1	Group C	0 (0%) 6.8 ± 4.6	0 (0%) 6.8 ± 4.6	4 (36%) 31.4 ± 45.1 ^†^	1 (9%) 25.5 ± 45.1	1 (9%) 24.1 ± 45.5
	Group F	0 (0%) 7.7 ± 4.7	0 (0%) 6.4 ± 2.3	5 (45%) 45.9 ± 58.1 ^†^	5 (45%) 31.4 ± 27.8 ^†^	2 (18%) 21.4 ± 22.0
A/H3N2	Group C	0 (0%) 6.8 ± 4.6	0 (0%) 6.8 ± 4.6	1 (9%) 13.6 ± 10.7 ^†^	1 (9%) 14.1 ± 10.4 ^†^	1 (9%) 12.7 ± 10.6
	Group F	0 (0%) 10.5 ± 6.5	0 (0%) 10.0 ± 6.7	1 (9%) 14.5 ± 10.8	2 (18%) 15.9 ± 13.6	1 (9%) 14.5 ± 10.8
B	Group C	0 (0%) 11.7 ± 6.6	0 (0%) 10.6 ± 5.8	2 (22%) 17.8 ± 13.0	2 (22%) 17.8 ± 13.0	1 (11%) 13.9 ± 10.5
	Group F	0 (0%) 8.0 ± 4.8	0 (0%) 8.5 ± 6.3	1 (10%) 13.5 ± 23.5 *	1 (10%) 14.0 ± 23.3 *	1 (10%) 10.0 ± 10.8

The upper number is number of subjects with a seroprotective antibody titer and the seroprotective rate (%) which is presented within parenthesis. The lower number is mean ± SD of the HI antibody titers. Analysis of the number (rate (%)) of seroprotective patients and HI antibody titers were performed in the seronegative subjects (the titer < 40) before vaccination (at week 4). Because there were three subjects (group C: two subjects; group F: one subject) with seroprotective antibody titers against B antigen (the titer ≥ 40) before vaccination, these subjects were excluded from analysis of the number (rate (%)) of seroprotective patients and HI antibody titers against B antigen. No subjects had seroprotective HI antibody titers against A/H1N1 or A/H3N2 antigen before vaccination. Seroprotective rate (%) was calculated as follows: Seroprotective rate (%) = 100 × (Number of seroprotected subjects)/(Number of seronegative subjects before vaccination). * Significant differences between groups C and F (*p* < 0.05). ^†^ Significant differences compared with week 4 in each group (*p* < 0.05).

The upper number in Table 6 shows the number of subjects with seroprotective antibody titers and seroprotective rate (%) which is presented within parenthesis. Although few subjects with seroprotective antibody titers against A/H3N2 and B after vaccination were found in either group in this study, the number of those with seroprotective titers against A/H1N1 antigen increased at week 6. The increased number of subjects with seroprotective titers against A/H1N1 antigens in group F was maintained at week 8, whereas the number of those with seroprotective titers in group C decreased rapidly at week 8. There were no significant differences in the number of patients with the seroprotective titers against A/H1N1, A/H3N2, or B antigens between the two groups.

Changes in specific HI antibody titers against the influenza vaccine are shown in Table 6 (lower numbers). The HI antibody titers against the A/H1N1 antigens in group F increased significantly at weeks 6 and 8 compared with those at week 4 (the time of vaccination). In contrast, the antibody titers against the A/H1N1 antigens in group C were augmented significantly at week 6 compared with those at vaccination, but not at week 8. There were no significant differences in the antibody titers against A/H1N1 antigens between the two groups. The antibody titer to B antigen was significantly lower for group F subjects than group C subjects at weeks 6 and 8.

## 4. Discussion

We examined the effects of an enteral formula containing two different types of prebiotics, BGS and GOS, and fermented milk products on intestinal microbiota and immunological response after influenza vaccination in enterally fed elderly patients in a randomized, controlled trial. Our results revealed that enteral administration of the study formula containing prebiotics and fermented milk products may improve intestinal microbiota and may contribute to maintenance of antibody titers against influenza viral A/H1N1 antigen for longer periods in elderly patients, without any change in blood nutritional indices.

The study formula contains almost the same nutrients as the standard formula, except for selenium, manganese, vitamin A and vitamin C (Table 1). The previous study in institutionalized elderly subjects showed that the simultaneous administration of micronutrients, such as selenium, manganese, vitamin A and vitamin C, did not increase the antibody response after influenza vaccination [20]. A different study of elderly subjects also suggested that the consumption of vitamin C had no effect on HI antibody titers against influenza vaccine antigens [21]. No consistent associations were found between vitamin C levels and immune responses to vaccine antigens in undernourished children [22]. Together, these results suggest that the differences in selenium, manganese, vitamin A, and vitamin C levels in formula given to subjects in groups C and F most likely did not affect antibody titers against influenza vaccine in our study.

We measured the counts of seven types of fecal bacteria in elderly patients administered formula by PEG. These bacterial counts observed in this study were almost the same as previous reports, except for *Bifidobacterium* [16,18,23,24]. The mean *Bifidobacterium* counts in this study in groups C (4.4 ± 3.4) and F (5.6 ± 2.7) (Table 5) at week 0 were about 1/10,000 of those (9.5 ± 0.1) [18] and (9.6 ± 0.5) [23] from previous studies of healthy elderly subjects. In addition, the standard deviations of *Bifidobacterium* counts in groups C and F were larger than those in reported previous studies in healthy elderly subjects [18,23]. These findings indicate that the individual variation of fecal *Bifidobacterium* counts in elderly patients administered formula by PEG was larger than those in the healthy elderly population.

In this study, enteral administration of the study formula containing prebiotics and fermented milk products significantly increased fecal *Bifidobacterium* counts. Therefore, our results suggest that enteral feeding of the study formula may improve microbial imbalance, termed dysbiosis, in enterally fed patients.

Except for *Bifidobacterium*, there were few significant differences between the two groups regarding the bacterial counts. Though *C. coccoides* counts were significantly decreased in group F at week 12 (*p* < 0.05), there was little difference in the average value between the two dietary groups. In addition, there were no significant differences in *C. coccoides* counts in groups C and F at week 12 compared with week 0 (*p* > 0.05). These results suggest that administration of the study formula does not affect intestinal *C. coccoides* counts.

Administration of prebiotics such as GOS is effective in increasing the population of bifidobacteria [25]. A new type of prebiotics, BGS, was also found to stimulate growth of bifidobacteria in the colon [11,26]. In fact, BGS intake was effective to increase the detection rate of fecal bifidobacteria and improved the fecal properties in enterally fed elderly patients [11]. Although probiotics are generally defined as live microbial feed supplements, heat-killed *Lactobacillus* administration also exhibits a stimulatory effect on bifidobacteria [27]. Therefore, the three nutritional ingredients of the study formula, namely, BGS, GOS, and heat-treated milk products fermented by *L. delbrueckii* subsp. *bulgaricus* and *S. thermophilus*, may additively or synergistically improve the imbalance of microflora in enterally fed elderly patients.

Few subjects in this study attained seroprotective titers against A/H3N2 and B antigens after influenza vaccination in either experimental group, though antibody titers against A/H1N1 were enhanced. On the other hand, our previous results showed that HI antibody titers against A/H1N1 and A/H3N2 antigens were increased after vaccination with little enhancement of titers against B antigens [12]. There were differences in antibody responses to the A/H3N2 antigen between our previous study and our present study. Influenza vaccines used in both studies contained different influenza A/H3N2 strain antigens, A/Hiroshima/52/2005 in our previous study and A/Victoria/210/2009 in our present study. A consecutive four-year study in influenza-vaccinated healthy elderly subjects suggested that antibody responses to the A/H3N2 antigen changed due to the use of influenza vaccines containing different influenza A/H3N2 strain antigens [28]. Therefore, the difference of A/H3N2 strain between both studies may have affected the HI antibody responses.

In our present study, influenza vaccination augmented the seroprotective rates and HI antibody titers against A/H1N1 antigens in both groups. The HI antibody titers in group F against the A/H1N1 antigens increased significantly at weeks 6 and 8 compared with the baseline, though in group C the antibody titers against the A/H1N1 antigens increased significantly at week 6 only. The seroprotective rate also increased at weeks 6 and 8 in group F, whereas an augmented seroprotective rate in group C was observed at week 6 only. These results indicated that the enteral administration of the study formula containing prebiotics and fermented milk products maintained enhanced antibody titers for longer periods in elderly individuals. In undernourished elderly persons, vaccines may induce a lower level of protection than in well-nourished persons [13]. Although, in this study, there were significant differences in Alb levels between the two dietary groups at week 12, no differences in serum Alb levels between the two dietary groups were found at weeks 6 and 8. Therefore, increased serum Alb levels at week 12 in group F are considered not to affect the augmented antibody titers at weeks 6 and 8 after influenza vaccination. In addition, daily intake of energy, protein, fat and carbohydrates, and serum levels of nutrition indices (TP, p-Alb, and Tf) during study period were similar in groups C and F. These results demonstrated that differences in the nutritional status of subjects in both groups did not affect immune responses after vaccination.

Influenza is a leading cause of hospitalization and death for individuals over 75 years of age [29]. Influenza vaccination protects against influenza illness and reduces the risk of death and hospitalization associated with serious complications, such as pneumonia [2,3]. Our results showed that enteral intake of the study formula containing two types of prebiotics and fermented milk products may maintain augmented HI antibody titers against influenza vaccine antigens during longer periods in elderly patients. These results suggest that the administration of formula containing two types of prebiotics and fermented milk products may reduce the risk of death and hospitalization caused by influenza virus infection among the elderly vaccinated against influenza.

The limitation of this study is that the intervention was conducted only among elderly patients administered formula by PEG, suggesting it is hard to apply the results from the present study to a healthy subjects. The strengths of our study include the use of the enteral formula containing prebiotics and heat-treated fermented milk products. Severely immunodeficient subjects may be at risk from treatment with live probiotic microorganisms [30] and therefore the use of prebiotics or nonviable bacteria would be a safer alternative.

## 5. Conclusions

Our results show that enteral administration of the study formula containing two types of prebiotics and fermented milk products may improve intestinal microbiota and that enteral administration of the study formula may maintain antibody titers against influenza viral A/H1N1 antigens during longer periods in elderly patients. Interestingly, in a study in infants, who are in general as vulnerable as the elderly, consumption of fermented formula and increased antibody responses after vaccination correlated with fecal *Bifidobacterium* levels [31]. Especially, when taking this past study into consideration, our results suggest that administration of the study formula maintains antibody titers against influenza viral A/H1N1 antigens for longer periods through the improvement of intestinal microflora.

## References

[1] Fleming D.M., Elliot A.J. (2005). The impact of influenza on the health and health care utilisation of elderly people. Vaccine.

[2] Nichol K.L., Margolis K.L., Wuorenma J., Von Sternberg T. (1994). The efficacy and cost effectiveness of vaccination against influenza among elderly persons living in the community. New Engl. J. Med..

[3] Vu T., Farish S., Jenkins M., Kelly H. (2002). A meta-analysis of effectiveness of influenza vaccine in persons aged 65 years and over living in the community. Vaccine.

[4] Chen W.H., Kozlovsky B.F., Effros R.B., Grubeck-Loebenstein B., Edelman R., Sztein M.B. (2009). Vaccination in the elderly: An immunological perspective. Trends Immunol..

[5] Vijaya Kumar S.G., Singh S.K., Goyal P., Dilbaghi N., Mishra D.N. (2005). Beneficial effects of probiotics and prebiotics on human health. Pharmazie.

[6] Bengmark S. (2003). Use of some pre-, pro- and synbiotics in critically ill patients. Best Pract. Res. Clin. Gastroenterol..

[7] Bunout D., Barrera G., Hirsch S., Gattas V., de la Maza M.P., Haschke F., Steenhout P., Klassen P., Hager C., Avendaño M., Petermann M., Muñoz C. (2004). Effects of a nutritional supplement on the immune response and cytokine production in free-living Chilean elderly. JPEN J. Parenter. Enteral Nutr..

[8] Vos A.P., Haarman M., van Ginkel J.W., Knol J., Garssen J., Stahl B., Boehm G., M’Rabet L. (2007). Dietary supplementation of neutral and acidic oligosaccharides enhances Th1-dependent vaccination responses in mice. Pediatr. Allergy Immunol..

[9] Boge T., Rémigy M., Vaudaine S., Tanguy J., Bourdet-Sicard R., van der Werf S. (2009). A probiotic fermented dairy drink improves antibody response to influenza vaccination in the elderly in two randomised controlled trials. Vaccine.

[10] Hojo K., Yoda N., Tsuchita H., Ohtsu T., Seki K., Taketomo N., Murayama T., Iino H. (2002). Effect of ingested culture of *Propionibacterium freudenreichii* ET-3 on fecal microflora and stool frequency in healthy females. Biosci. Microflora.

[11] Seki K., Nakao H., Umino H., Isshiki H., Yoda N., Tachihara R., Ohuchi T., Saruta H., Suzuki K., Mitsuoka T. (2004). Effects of fermented milk whey containing novel bifidogenic growth stimulator produced by *Propionibacterium* on fecal bacteria, putrefactive metabolite, defecation frequency and fecal properties in senile volunteers needed serious nursing-care taking enteral nutrition by tube feeding. Chonaisaikingaku zasshi (Japanese).

[12] Akatsu H., Nagafuchi S., Kurihara R., Okuda K., Kanesaka T., Ogawa N., Kanematsu T., Takasugi S., Yamaji T., Takami M. (2015). Enhanced vaccination effect against influenza by prebiotics in elder-patients receiving enteral nutrition. Geriatr. Gerontol. Int..

[13] Lesourd B.M. (1997). Nutrition and immunity in the elderly: Modification of immune responses with nutritional treatments. Am. J. Clin. Nutr..

[14] Kojimahara N., Maeda A., Kase T., Yamaguchi N. (2006). Cross-reactivity of influenza A (H3N2) hemagglutination-inhibition antibodies induced by an inactivated influenza vaccine. Vaccine.

[15] Scharpé J., Peetermans W.E., Vanwalleghem J., Maes B., Bammens B., Claes K., Osterhaus A.D., Vanrenterghem Y., Evenepoel P. (2009). Immunogenicity of a standard trivalent influenza vaccine in patients on long-term hemodialysis: An open-label trial. Am. J. Kidney Dis..

[16] Matsuki T., Watanabe K., Fujimoto J., Takada T., Tanaka R. (2004). Use of 16S rRNA gene-targeted group-specific primers for real-time PCR analysis of predominant bacteria in human feces. Appl. Environ. Microbiol..

[17] Dubernet S., Desmasures N., Guéguen M. (2002). A PCR-based method for identification of lactobacilli at the genus level. FEMS Microbiol. Lett..

[18] Bartosch S., Fite A., Macfarlane G.T., McMurdo M.E. (2004). Characterization of bacterial communities in feces from healthy elderly volunteers and hospitalized elderly patients by using real-time PCR and effects of antibiotic treatment on the fecal microbiota. Appl. Environ. Microbiol..

[19] Rinttilä T., Kassinen A., Malinen E., Krogius L., Palva A. (2004). Development of an extensive set of 16S rDNA-targeted primers for quantification of pathogenic and indigenous bacteria in faecal samples by real-time PCR. J. Appl. Microbiol..

[20] Allsup S.J., Shenkin A., Gosney M.A., Taylor S., Taylor W., Hammond M., Zambon M.C. (2004). Can a short period of micronutrient supplementation in older institutionalized people improve response to influenza vaccine? A randomized controlled trial. J. Am. Geriatr. Soc..

[21] Crogan N.L., Velasquez D., Gagan M.J. (2005). Testing the feasibility and initial effects of iron and vitamin C to enhance nursing home residents’ immune status following an influenza vaccine. Geriatr. Nurs..

[22] Moore S.E., Goldblatt D., Bates C.J., Prentice A.M. (2003). Impact of nutritional status on antibody responses to different vaccines in undernourished Gambian children. Acta Paediatr..

[23] Benno Y., Endo K., Mizutani T., Namba Y., Komori T., Mitsuoka T. (1989). Comparison of fecal microflora of elderly persons in rural and urban areas of Japan. Appl. Environ. Microbiol..

[24] Tuohy K.M., Kolida S., Lustenberger A.M., Gibson G.R. (2001). The prebiotic effects of biscuits containing partially hydrolysed guar gum and fructo-oligosaccharides—A human volunteer study. Br. J. Nutr..

[25] Moro G., Arslanoglu S., Stahl B., Jelinek J., Wahn U., Boehm G. (2006). A mixture of prebiotic oligosaccharides reduces the incidence of atopic dermatitis during the first six months of age. Arch. Dis. Child..

[26] Uchida M., Mogami O., Matsueda K. (2007). Characteristic of milk whey culture with *Propionibacterium freudenreichii* ET-3 and its application to the inflammatory bowel disease therapy. Inflammopharmacology.

[27] Rigon-Zimmer K., Mullie C., Tir-Touil-Meddah A., Buisson P., Leke L., Canarelli J.P. (2008). Impact of colostomy on intestinal microflora and bacterial translocation in young rats fed with heat-killed *Lactobacillus acidophilus* strain LB. Folia Microbiol. (Praha).

[28] Murasko D.M., Bernstein E.D., Gardner E.M., Gross P., Munk G., Dran S., Abrutyn E. (2002). Role of humoral and cell-mediated immunity in protection from influenza disease after immunization of healthy elderly. Exp. Gerontol..

[29] Goodwin K., Viboud C., Simonsen L. (2006). Antibody response to influenza vaccination in the elderly: A quantitative review. Vaccine.

[30] Adams C.A. (2010). The probiotic paradox: Live and dead cells are biological response modifiers. Nutr. Res. Rev..

[31] Mullié C., Yazourh A., Thibault H., Odou M.F., Singer E., Kalach N., Kremp O., Romond M.B. (2004). Increased poliovirus-specific intestinal antibody response coincides with promotion of *Bifidobacterium longum-infantis* and *Bifidobacterium*
*breve* in infants: A randomized, double-blind, placebo-controlled trial. Pediatr. Res..

